# Historical Transition of Eco-Structure in a Tidal Flat Caused by Expansion of Sewerage Treatment Area

**DOI:** 10.1100/tsw.2004.29

**Published:** 2004-05-11

**Authors:** Hideki Tatsumoto, Yuichi Ishii, Motoi Machida, Kazuo Taki

**Affiliations:** Department of Material Technology, Chiba University, 1-33, Yayoi-cho, Inage-ku, Chiba-Shi, Chiba-ken, 263-8522, Japan; ^2^ Graduate School of Science and Technology, Chiba University, 1-33, Yayoi-cho, Inage-ku, Chiba-shi, Chiba-ken, 263-8522, Japan; ^3^ Department of Life and Environmental Sciences, Chiba Institute of Technology, 2-17-1, Tsudanuma, Narashino-shi, Chiba-ken, 275-8588, Japan

**Keywords:** lagoon type tidal flat, urban coastal zone, Ulva spp., sewerage system, ecosystem structure, environmental dynamics, chaos ecosystem

## Abstract

An artificial tidal flat was prepared for the mitigation tool on coastal environment. However, it is considered that most of the flat was not restored to the sufficient amenities for aquatic living things, migratory birds, etc. because none of the ecological mechanisms were understood or planned for. It is therefore investigated in this paper that historical transition factors in ecosystem structure are selected and traced with the diffusion of a public sewerage system, and with environmental factors such as water quality, sediment condition, and aquatic producers in the Yatsu Tidal Flat. As a result, it can be defined that the tidal flat, just like a lagoon, was formed artificially with reclamation and development of its circumference at the first step of transition; the water quality and sediment condition gradually became brackish water and muddy sediment conditions, interactively. The ecosystem pyramid forming orderly layers according to trophic level appeared as a high-bio-production potential in its tidal flat. In the second step, i.e., in recent years, the characteristics of water quality and sediment conditions evolved into a foreshore tidal flat, namely, conditions in the flat observed were that the progression of water included a high concentration of chloride ion as seawater and sediment conditions became sandy. Because of that, the inflowing fresh water and organic mater from the land area decreased with the improvement of the public sewerage system. The ecosystem pyramid was distorted into a chaos pyramid, with inversion of *Ulva* spp.

## INTRODUCTION

A tidal flat is, generally, located in a boundary zone between land and sea. The environmental factors are therefore, drastically changed with periodic characteristics of tide action and fresh water supplied from rivers. The growth rate of algae, such as diatoms and/or flagellums forming biofilm on the surface of the sediment layer, is kept at a very high level because of the continuity nutrient salt supply from areas, land, and sea. Then, the diversity according to floristic composition, namely the tidal food chain ecosystem, is established in the area by the addition of feed for fish and birds. On the other hand, the tidal flat is a very important place for human living as symbiosis between human living and natural environment. Although the area has been decreased by the reclamation of shallow sea with a view to expanding the economical field, it has been recently recognized that the tidal flat as a wetland is important for the key area of the natural food chain. The "Convention on Wetlands (Ramsar Iran, 1971)", whose purpose is to preserve a migratory area, was adapted by the International Union for Conservation of Nature and Natural Resources (IUCN) in 1971. Thirteen wetland areas in Japan were registered for the convention at the end of 2002.

Yatsu tidal flat, which was registered under the Convention on Wetlands in June 1993, is located in the urban coastal zone of Tokyo Bay, as shown in Fig. 1, and has an extremely unique process on the transition of tidal function, including tidal geomorphic development. The tidal flat had a characteristic of vast foreshore type observed at the front of the coastline in Tokyo Bay until 1971, i.e., until about 30 years before. After the reclamation works, the utilization type of surrounding shoreline was changed from fishery and intimacy of water areas to industrial and residential areas. Yatsu tidal flat (area: 40.1 ha) was finally left alone in shallow seawater and its water shoreline distorted into a lagoon-type tidal flat in shape, by concrete shore protections[1,2].

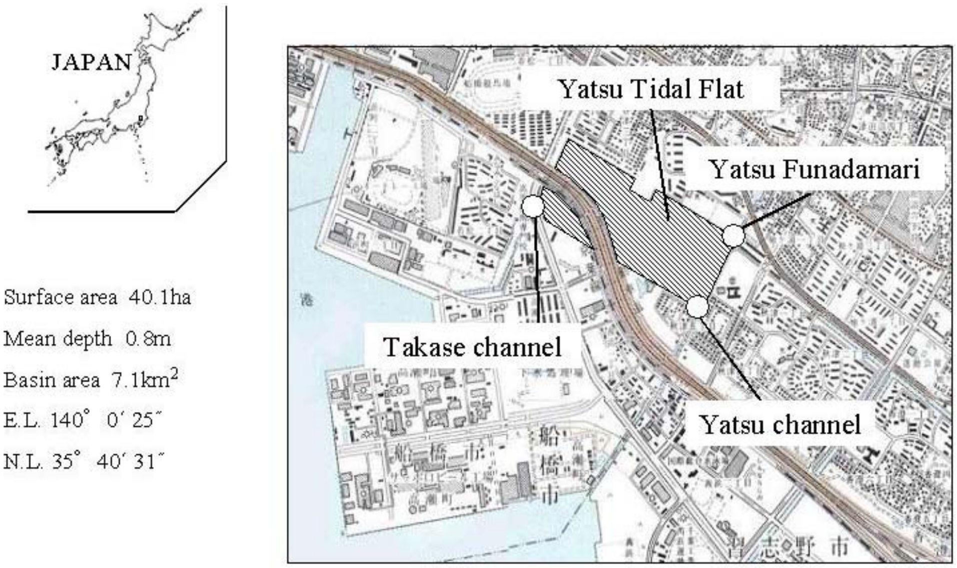

**FIGURE 1**. Location of Yatsu tidal flat and sampling sites.

The Yatsu tidal flat has been utilized as an eating and resting space for migratory birds, which move a long distance between Siberia in the Far Eastern Area and Australia in the Southern Hemisphere every year. For this reason, the Yatsu tidal flat was registered under the Convention on Wetlands in June 1993. A decrease of macrobenthos as food for migratory birds was caused by a decrease of mud sediment areas with organic matter inflowing from the surrounding area. The significance of the existence of the Yatsu tidal flat was furthermore threatened by the extinction of macrobenthos and by the decrease of the resting space because of extraordinary growth of *Ulva* spp. in the tidal flat.

In this paper, it was investigated that the transition of the ecosystem in the Yatsu tidal flat was induced by the diffusion of a public sewerage system, especially in water quality and sediment conditions, which were analyzed with various data published by administrative organizations.

## ENVIRONMENTAL FACTORS IN YATSU TIDAL FLAT

The major factors for the environmental ecosystem structure, i.e., ecological pyramid in a tidal flat, are generally considered to be water quality, sediment condition, topography characteristics, tide action, and living things[1]. Here, the basin area of the Yatsu tidal flat is administratively divided in two cities: Narashino City and Funabashi City.

The transitions of water quality and sediment conditions in the tidal flat are observed as dependent phenomena with the diffusion of the public sewerage system, as shown in Figs. 2, 3, and Table 1. The data regarding $Cl^-$ concentration in Fig. 3 and various kinds of data in Table 1 are obtained from the Environmental Agency of Japan[3], etc. Fig. 2 is drawn from the data published in the White Papers regarding Narashino City Statistics (1984–1996)[4] and Funabashi City Statistics (1984–1996)[5]. The production loads shown in Fig. 2 represent the amount of total loads produced in the drainage basin of the Yatsu tidal flat and the inflow loads represent the amount of loads that flowed into the tidal flat from its basin. The popularization rate of sewerage means total popularization rate of the public sewerage system in both cities. The discharge from the sewage system into the flat decreased year by year to 16.8% during the 12 years from 1983–1995. Chloride ion concentration is, therefore, observed to be an increasing trend. In other words, the increase of chloride ion concentration in the flat means the phenomena of a change in water quality from freshwater to seawater.

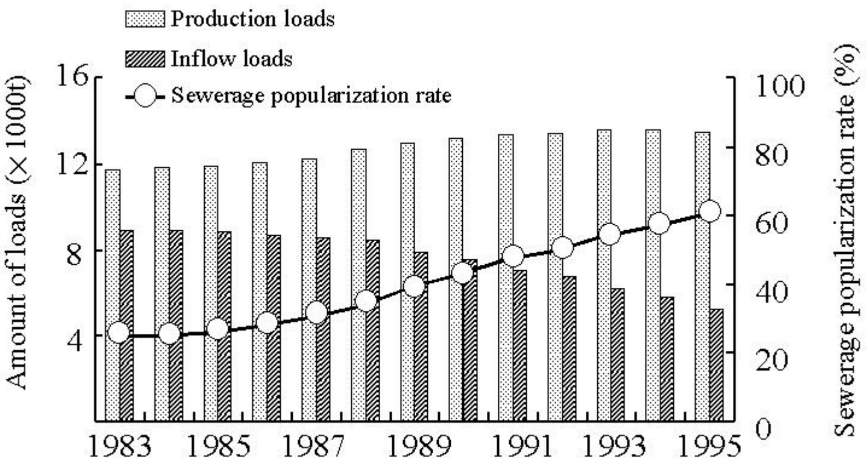

**FIGURE 2**. Change of popularization of sewerage and amount of loads.

Fig. 3 shows series data on the chloride ion concentration corresponding to sewerage diffusion rate at the monitoring points of Yatsu channel, Takase channel, and Yatsu-Funadamari, which are located in the flat and shown as a symbol of circle line in Fig. 1. Fig. 3 is drawn from the series set data, which are sewerage popularization rate and $Cl^-$ concentration. These three monitoring points show the same increasing tendency. However, from the data obtained at Takase monitoring point, the increasing tendency is not indicated as clearly as at other points because the treated water from the sewerage plant flows out at the river mouth of Takase channel.

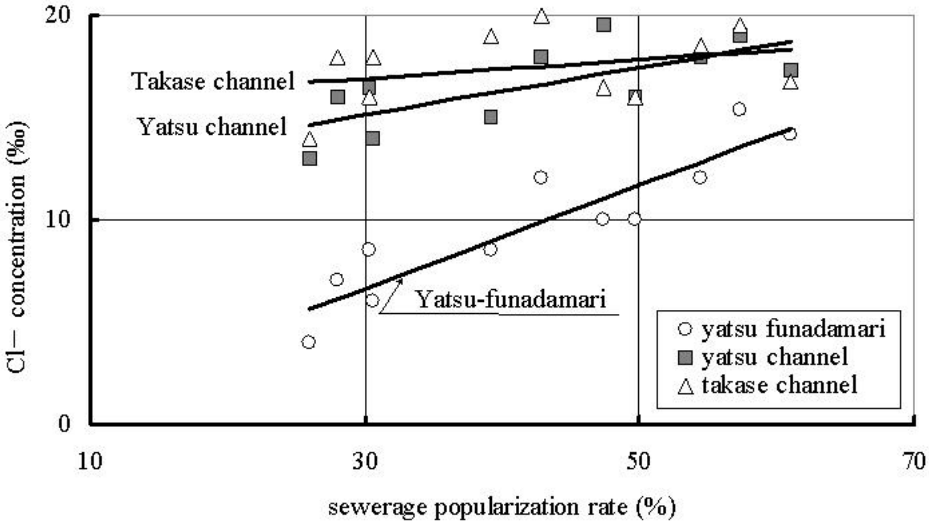

**FIGURE 3**. Relationship between sewerage popularization rate and $Cl^-$ concentration.

**TABLE 1**
**Comparison of Sediment Conditions**

| | 1984 | 1995 |
|---|---|---|
| depth of mud layer (cm) | 25 - 200 | 0 - 50 |
| I.L. (%) | 3.6 - 11 | 2.1 - 6.9 |
| sand area (ha) | 4 | 4 |
| sand -mud area (ha) | 10 | 18 |
| mud area (ha) | 20 | 15 |
| gut area (ha) | 6 | 2 |

On the sediment condition as shown in Table 1, which sorts the information of various data, the mud area and gut area in the flat have lost 25% from 20–15 ha and 67% from 6–2 ha, respectively, and then sandy-mud area has increased 80% from 10–18 ha. Ignition Loss (i.e., I.L.) of sediment in the flat has been decreased because of the decreasing of nutrient salts that are directly supplied from domestic wastewater in the river basin. Moreover, the reformation on the depth of mud layer, from 25–200 cm in 1984 to 0–50 cm in 1995 is very large in the case of the Yatsu tidal flat. It seems to be changing very rapidly from a mud layer to a sand layer, as the mud is washed out into the outside of the coastal zone in Tokyo Bay.

In that case, a structure was built in the tidal flat, such that the eco-environmental system is able to change from the artificial condition to the quash-original or rebounded condition with the changing of inflows, such as hinterland conditions, e.g., the diffusion of sewerage treatment in catchments area and the changing of life style in the area. By way of parenthesis, it appears that the characteristics of seawater is increased such as the observation of extraordinary growth of macroalgae, with decreasing of inflow discharge from hinterland.

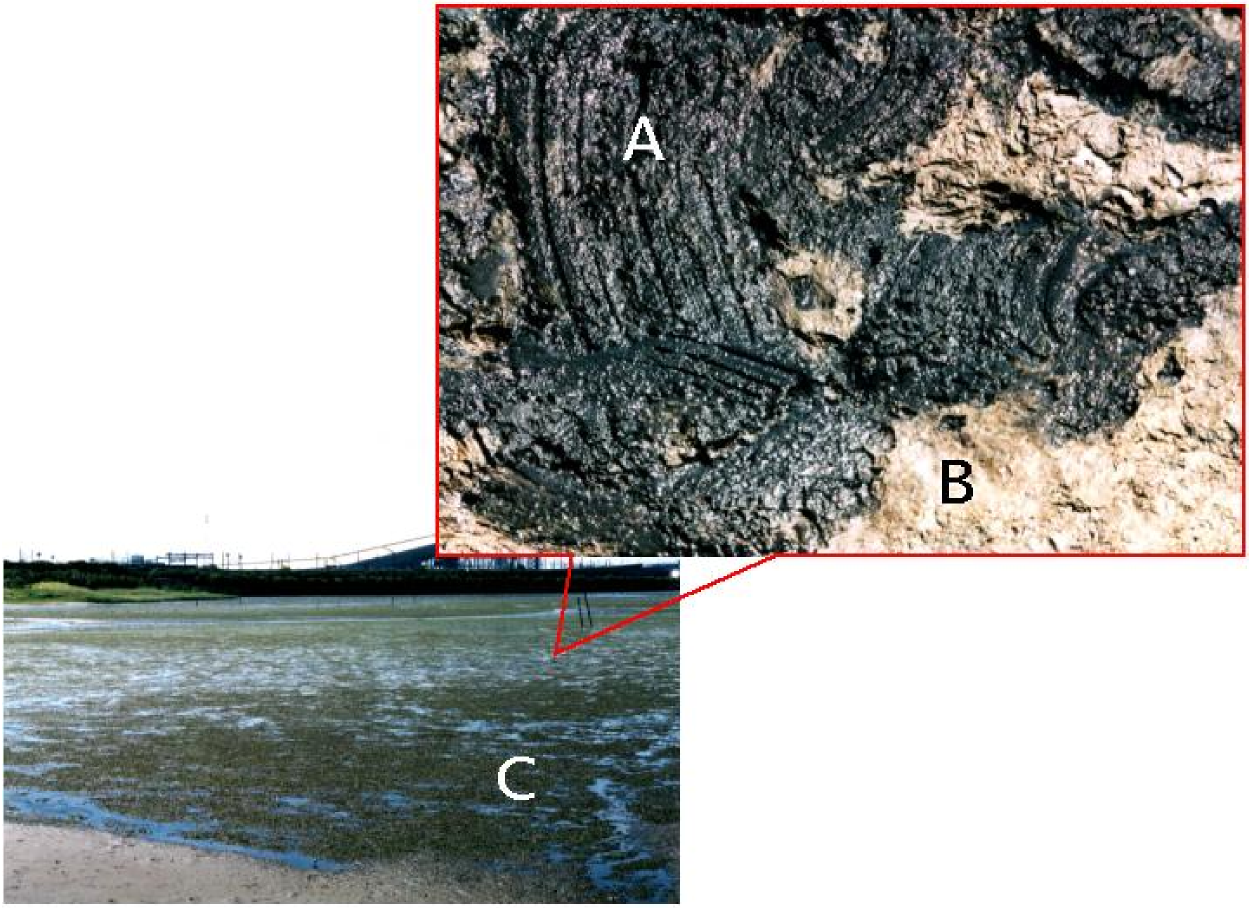

**Photo.1** Reduction state sediment covered with *Ulva* spp. (A) shows the sediment under *Ulva* colony. (B) shows Ulva spp. decayed on sediment. (C) shows *Ulva* spp. habited in Yatsu tidal flat.

## INVASION OF MACROALGAE

In recent years, it has been observed that *Ulva* spp., identified as *U. pertusa* and *U. japonica*, has become overgrown in this tidal flat, and that the growth area has increased year by year. *Ulva* spp. is generally classified in one species group of large green algae[6], and is observed in the flat of sand and/or sandy-mud sediment condition rather than mud-sediment condition. However in 1984, which was the first year of environmental monitoring, the *Ulva* spp. was not observed. It was recognized in an area of 6.8 ha in 1995, 13 ha in 1999, and over 20 ha in 2000. Finally, in the summer of 2001, nearly two-thirds of the area of this tidal flat became occupied by *Ulva* spp. It should be considered that the extraordinary growth of these algae is caused by sufficient nutrient salts dissolved in the water and by the salt concentration increasing due to penetration into the flat, and by the expansion of the sandy-mud sediment area, namely, the reasons are increasing seawater/fresh water ratio and sand/mud ratio.

Of the sediment condition in Yatsu tidal flat, it is considered that *Ulva* spp. decayed and made anaerobic sediment state after its extraordinary growth. The state of oxidation-reduction potential (ORP) in the sediment layer thickly covered with *Ulva* spp. is shown as the reduced layer colored in black in Photo 1, which is in high anaerobic condition as a value between –200 and –250 mV. In such a reduction area, none of the macrobenthos such as *Annelida* and bivalves was observed, which support a tidal flat ecosystem as main predator. In comparison between the macrobenthic fauna in 1984 and in 1995, the total species number of macrobenthos increased from 13 to 39[3], however, the total number of individuals clearly decreased, and no living points appeared in the sediment layer covered with *Ulva* spp.

It is suggested as a result, that biological diversity has been changing from “large quantity of small species” condition to “small quantity of large species” condition, because of the sediment changing from mud to sand/sandy-mud; namely, it means the “quality changing” from stabilized state to chaos state according to ecosystem in Yatsu tidal flat with brittle bio-production.

## TRANSITION MECHANISMS ON ECOSYSTEM STRUCTURE OF YATSU TIDAL FLAT

The ecosystem based on a food chain is formed also in Yatsu tidal flat which is classified as an artificial lagoon-type tidal flat and is operated by the tide with only two narrow channels connected to Tokyo Bay. Before *Ulva* spp. appeared in the flat, i.e., until 1994, eutrophicated water quality composed with nitrogen and phosphorus was estimated in a high concentrated condition, and monitored *Cheatceros* sp. and *Skeletonema* sp. in plankton, *Nitzschia* sp. in attached algae, *Neanthes succinea* and *Tylorrhhynchus heterochaetus* in macrobenthos (Furota and Suzuki, 1999), and *Charadrius alxandrinus* and *Clidris alpina* [8] in birds in Table 2. Since fishes are excursion nature, they are not shown in a table. It is considered with 1986 and 1994 that dominant species which are come in Yatsu tidal flat are the same species.. These kinds of biota established a common style structure on the food chain, as an ecosystem pyramid shown in Fig. 4 (a). Namely, an orderly layer ecosystem pyramid on a trophic level — such as phytoplankton and attached algae in producer, zooplankton in first predator, macrobenthos in second predator, and birds in final predator — had appeared as a high-bio-production potential in its tidal flat.

In 1995, the rooting *Ulva* spp. was observed for the first time in the area of 6.8 ha as sandy sediment area. This *Ulva* spp. was a new producer for the flat, which was not observed in the field monitoring in 1986. It is considered that all photosynthesis with phytoplankton and attached algae turned out to be cut, if *Ulva* spp. as producer level invaded into the flat, because the leaves of *Ulva* spp. can easily cover the sediment surface and shield the irradiation of sunlight for photosynthetic biota. As a result, the occupation rate in the producer level — that has been kept by phytoplankton — decreases gradually.

As shown in Fig.4 (b) and Table 2, only two species, *Batillaria cumingii* (snail) classified in macrobenthos and *Anas penelope* (mallard) in birds, are discovered as the predator against *Ulva* spp.

**TABLE 2**
**Dynamics of Biota in Yatsu Tidal Flat**

| | | 1986 | 1995 |
|---|---|---|---|
| 3rd predator------- | Birds | *Charadrius alexandrinus*<br>*Calidris alpina* | ***Anas penelope***<br>*Charadrii* spp. |
| 2nd predator------ | Macrobenthos | *Neanthes succinea*<br>*Tylorrhhynchus heterochaetus* | ***Batillaria cumingii***<br>*Ruditapes philippinarum* |
| 1st predateor ----- | Zooplankton | *Oithona davisae*<br>*Nectochaeta* of POLYCHAETA | *Oithona davisae*<br>*Nectochaeta* of POLYCHAETA |
| Produsors ----- | Phytoplankton | *Skeletonema costatum* | *Cheatceros* spp.<br>*Skeletonema costatum* |
| | Attached algae | *Nitzschia* spp. | *Nitzschia* spp. |
| | Macroalgae | —— | ***Ulva* spp.** |

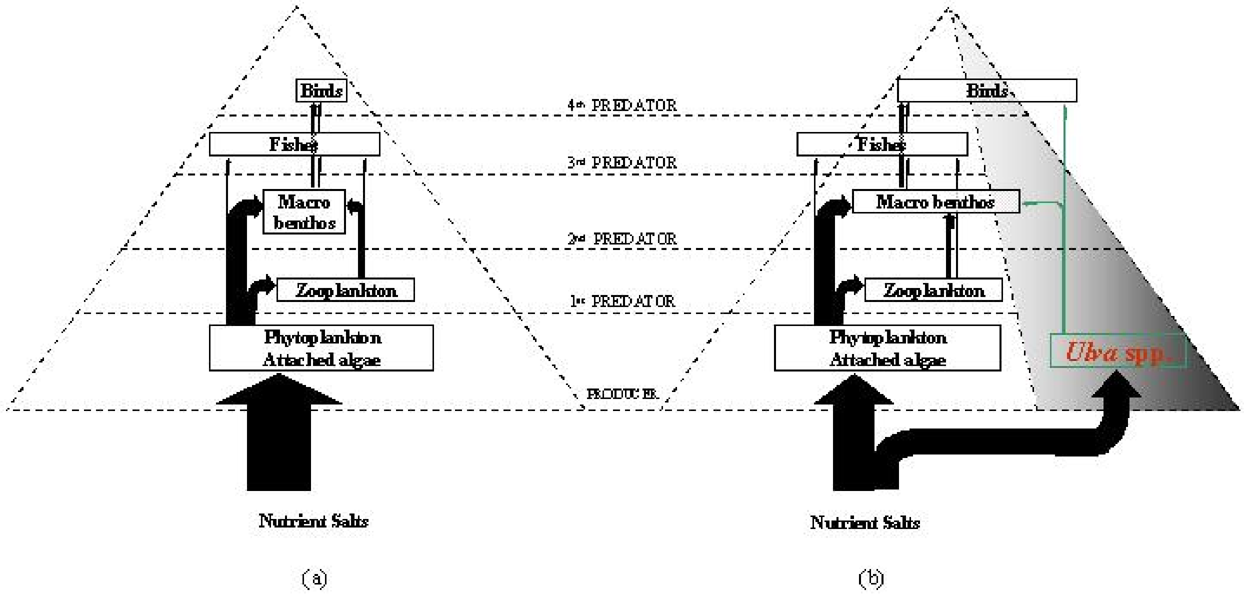

**FIGURE 4.** Transition of ecosystem structure on Yatsu tidal flat. The thickness of arrows is shown relative importance of nutrient salts fluxes.

Thus, it can be inferred with the monitoring results that the ecosystem of the Yatsu tidal flat is in transition and the state of the ecosystem is gradually changing from eutrophicated and a stabilized orderly state to a plural and chaos state, as the gradation in Fig.4 (b) shows. An arrow in Fig.4 (b) means the transitional increasing according to the occupied rate on *Ulva* spp. in the ecosystem pyramid, as ecological reorganization. Moreover, the distorted area induced by *Ulva* spp. is expanded in the ecosystem pyramid, and then the new predators in each trophic level are slowly produced level by level corresponding to new producers. The arrows in Fig.4 are shown relative importance of nutrient salts fluxes. The relative fluxes after invasion by *Ulva* spp. for phytoplankton and *Ulva* spp. were estimated about 2 and 1 respectively [9]. It can be verified with the monitoring data in Table 2 and Fig. 4 that the ecosystem pyramid is evolving into a new one as foreshore tidal flat year by year.

*Neanthes succinea* was observed to be the dominant species in the Yatsu tidal flat in 1986, the biomass sharply decreased from 90 N/0.1 $m^2$ (average value of five stations [7]) to 9.4 N/0.1 $m^2$ (average value of same stations as 1986) in 1995. It is, however, clearly defined that the species number of macrobenthos was increased from 19 species to 39 species, by the onsite-monitoring for 10 years. Transition of its biota is dependant on the increasing sandy area in the flat, i.e., a decrease of the inhabiting species in the muddy area and an increase of the species in sandy area, conversely. In other words, it is admitted that the inhabitant of *Ruditapes philippinarum* was increased from one monitoring station to two stations over 10 years from 1986 and the biomass changed from 5 to 7.5 N/0.1 $m^2$ (average value of two stations). It should be written as a special mention of the biota transition shown in Fig. 4 that

*Batillaria cumingii*, which did not exist heretofore, came to be observed with the occurrence of *Ulva* spp. in an ecosystem pyramid of the flat, and makes a first step for the diversity of biota in macrobenthos.

## CONCLUSIONS

Historical transition factors in ecosystem structure are selected in this paper and traced with the diffusion of a public sewerage system, and with environmental factors such as water quality, sediment condition and aquatic producers in Yatsu tidal flat. The results are summarized as follows:

1. It is defined that the water quality and the sediment condition in the flat changed from freshwater to seawater and from mud to sand, respectively, depending on the decreasing inflow loads caused by improvement of a public sewerage system. The state of oxidation-reduction potential (ORP) in sediment layer thickly covered with *Ulva* spp. is in reduced layer condition, which is in high anaerobic condition between –200 and –250 mV.
2. *Ulva* spp., identified as *U. pertusa* and *U. japonica*, became overgrown in this tidal flat. The flat sediment condition transferred from mud sediment to sand and/or sandy-mud sediment, and the growth area of *Ulva* spp. was observed on nearly two-thirds of the surface area of the flat.
3. None of macrobenthos such as *Annelida* and bivalves was observed in the reduction area, which support a tidal flat ecosystem as a main factor in second predator level. In comparison with macrobenthic fauna between 1984 and 1995, the total species number of macrobenthos has increased from 13 to 39. Individual total number in each species has, however, clearly decreased, and no living points appeared in sediment layer covered with *Ulva* spp.
4. It is suggested as a result, that biological diversity has been changing the condition from “large quantity of small species” to “small quantity of large species”, because of the sediment condition changing from mud to sand/sandy-mud; namely, it means the “quality changing” from stabilized state to chaos state according to ecosystem in Yatsu tidal flat with brittle bio-production.
5. The ecosystem structure in the Yatsu tidal flat is gradually changing from stabilized state to plural/chaos state. The adjusted new ecosystem in occupied rate of *Ulva* spp is expanding in the Yatsu tidal flat year by year, because of the extraordinary growth of *Ulva* spp.

## ACKNOWLEDGMENTS

This research was partially supported in 2001 and 2002 financial years by the grant from Research Institute of Chiba Institute of Technology.

## BIOSKETCHES

**Hideki Tatsumoto** is a Professor with the Department of Material Technology, Faculty of Engineering, Chiba University, Japan, and is an Environmental Scientist. He received B.Eng. degrees from Chiba Institute of Technology, Japan and Dr.Eng. degrees from Kyoto University, Japan.

**Yuichi Ishii** is a doctoral program student with the Graduate School of Science and Technology, Chiba University, Japan and is a Coastal Environmental Scientist. He received B.Eng. and M.Eng. degrees from Chiba Institute of Technology, Japan.

**Motoi Machida** is an Associate Professor with the Department of Material Technology, Faculty of Engineering, Chiba University, Japan and is an Environmental Chemist. He received B.S., M.S., and Dr.Eng. degrees from Hokkaido University, Japan.

**Kazuo Taki** is a Professor with the Department of Life and Environmental Sciences, Faculty of Engineering, Chiba Institute of Technology, Japan and is an Environmental Hydro-Scientist. He received B.Eng. degrees from Chiba Institute of Technology, Japan and M.Eng. and Dr.Eng. degrees from Chuo University, Japan.
